# Soil Carbon and Nitrogen Fractions and Crop Yields Affected by Residue Placement and Crop Types

**DOI:** 10.1371/journal.pone.0105039

**Published:** 2014-08-13

**Authors:** Jun Wang, Upendra M. Sainju

**Affiliations:** 1 College of Urban and Environmental Sciences, Northwest University, Xian, Shaanxi Province, China; 2 U.S. Department of Agriculture, Agricultural Research Service, Northern Plains Agricultural Research Laboratory, Sidney, Montana, United States of America; Institute for Sustainable Plant Protection, C.N.R., Italy

## Abstract

Soil labile C and N fractions can change rapidly in response to management practices compared to non-labile fractions. High variability in soil properties in the field, however, results in nonresponse to management practices on these parameters. We evaluated the effects of residue placement (surface application [or simulated no-tillage] and incorporation into the soil [or simulated conventional tillage]) and crop types (spring wheat [*Triticum aestivum* L.], pea [*Pisum sativum* L.], and fallow) on crop yields and soil C and N fractions at the 0–20 cm depth within a crop growing season in the greenhouse and the field. Soil C and N fractions were soil organic C (SOC), total N (STN), particulate organic C and N (POC and PON), microbial biomass C and N (MBC and MBN), potential C and N mineralization (PCM and PNM), NH_4_-N, and NO_3_-N concentrations. Yields of both wheat and pea varied with residue placement in the greenhouse as well as in the field. In the greenhouse, SOC, PCM, STN, MBN, and NH_4_-N concentrations were greater in surface placement than incorporation of residue and greater under wheat than pea or fallow. In the field, MBN and NH_4_-N concentrations were greater in no-tillage than conventional tillage, but the trend reversed for NO_3_-N. The PNM was greater under pea or fallow than wheat in the greenhouse and the field. Average SOC, POC, MBC, PON, PNM, MBN, and NO_3_-N concentrations across treatments were higher, but STN, PCM and NH_4_-N concentrations were lower in the greenhouse than the field. The coefficient of variation for soil parameters ranged from 2.6 to 15.9% in the greenhouse and 8.0 to 36.7% in the field. Although crop yields varied, most soil C and N fractions were greater in surface placement than incorporation of residue and greater under wheat than pea or fallow in the greenhouse than the field within a crop growing season. Short-term management effect on soil C and N fractions were readily obtained with reduced variability under controlled soil and environmental conditions in the greenhouse compared to the field. Changes occurred more in soil labile than non-labile C and N fractions in the greenhouse than the field.

## Introduction

Soil organic matter, as indicated by C and N levels, is an important component of soil quality and productivity. Increasing soil organic matter through enhanced C and N sequestration can also reduce the potentials for global warming by mitigating greenhouse gas emissions and N leaching by increasing N storage in the soil [1], [2]. Carbon and N sequestration usually occur when non-harvested crop residues, such as stems, leaves, and roots, are placed at the soil surface due to no-tillage [3], [4], [5]. Carbon and N sequestration rates, however, depend on the balance between the amounts of plant residue C and N inputs and rates of C and N mineralized in the nonmanured soil [6], [7]. Other benefits of increasing C and N storage include enhancement of soil structure and soil water-nutrient-crop productivity relationships [8].

Soil and crop management practices can alter the quantity, quality, and placement of crop residues in the soil, thereby influencing soil C and N storage, microbial biomass and activity, and N mineralization–immobilization [9], [10]. Residue placement in the soil under different tillage systems can influence C and N levels by affecting soil aggregation, aeration, and C and N mineralization [9], [11]. Crop types can affect the quantity and quality (C/N ratio) of crop residue returned to the soil and therefore on soil C and N levels [9], [12]. Legumes, such as pea, because of its higher N concentration and lower C/N ratio, decompose more rapidly in the soil and supply greater amount of N to succeeding crops than nonlegumes [12], [13]. As a result, N fertilization rates to crops following pea can be reduced to sustain yields [14], [15].

Because of large pool sizes and inherent spatial variability, soil organic C (SOC) and total N (STN) (slow or non-labile fractions) change slowly with management practices [16]. Therefore, measurements of SOC and STN alone may not adequately reflect changes in soil quality and nutrient status [16], [17]. Active (or labile) C and N fractions, such as potential C and N mineralization (PCM and PNM) that indicate microbial activity and N mineralization, and microbial biomass C and N (MBC and MBN) that refer to microbial biomass and N immobilization, change seasonally [16], [18]. Similarly, particulate organic C and N (POC and PON) that represent coarse organic matter and considered as intermediate C and N levels between slow and active fractions, provide substrates for microbes and influence soil aggregation [19], [20]. Available N fractions that influence plant growth and N losses due to leaching, denitrification, or volatilization are NH_4_-N and NO_3_-N [10], [12].

Although active C and N fractions in the soil can change more rapidly than the other fractions, these fractions sometime may not be readily changed within a crop growing season due to high variability in soil properties within a short distance in the field or in regions with limited precipitation, cold weather, and a short growing season [10], [12], [15]. Under controlled soil and environmental conditions, such as in the greenhouse, it may be possible to detect changes in these fractions more rapidly as affected by management practices than in the field. We hypothesized that surface placement of crop residue (a simulation of no-tillage in the field) under spring wheat can increase soil labile and non-labile C and N fractions and sustain crop yields compared to residue incorporation into the soil (a simulation of conventional tillage) under pea or fallow more in the greenhouse than in the field. Our objectives were to: (1) evaluate the effects of residue placement and crop types on crop yields, residue C and N losses, and soil labile and non-labile C and N fractions within a growing season in the greenhouse and the field and (2) determine if soil C and N fractions change more readily in the greenhouse than the field within a growing season.

## Materials and Methods

### Greenhouse experiment

The experiment was conducted under controlled soil and environmental conditions in the greenhouse with air temperatures of 25°C in the day and 15°C in the night. Soil samples were collected manually from an area of 5 m^2^ using a shovel to a depth of 20 cm under a mixture of crested wheatgrass [*Agropyron cristatum* (L.) Gaertn] and western wheatgrass [*Pascopyrum smithii* (Rydb.) A. Love] from a dryland farm site, 11 km east of Sidney, Montana, USA. The research farm site where soil samples were collected is under the management of USDA, Agricultural Research Service, Sidney, Montana and no endangered or protected species were involved or was negatively impacted by this research. The soil was a Williams loam (fine-loamy, mixed, frigid, Typic Argiborolls [International classification: Luvisols]) with 350 g kg^−1^ sand, 325 g kg^−1^ silt, 325 g kg^−1^ clay, 1.42 Mg m^−3^ bulk density, and 7.2 pH at the 0–20 cm depth. Soil C and N fractions in the sample before the initiation of the experiment are shown in Table 1. Soil was air-dried and sieved to 4.75 mm after discarding coarse organic materials and rock fragments. Eight kilograms of soil was placed in a plastic pot, 25 cm high by 25 cm diameter, above 3 cm of gravel at the bottom.

**Table 1:** Average soil organic C (SOC), total N (STN), particulate organic C and N (POC and PON), potential C and N mineralization (PCM and PNM), microbial biomass C and N (MBC and MBN), and NH_4_-N and NO_3_-N concentrations at the start of the experiment (n = 4).

Parameter	Concentration
SOC (g C kg^−1^)	11.80
POC (g C kg^−1^)	3.18
PCM (mg C kg^−1^)	9.25
MBC (mg C kg^−1^)	117.6
STN (g N kg^−1^)	1.29
PON (g N kg^−1^)	0.34
PNM (mg N kg^−1^)	8.95
MBN (mg N kg^−1^)	69.0
NH_4_-N (mg N kg^−1^)	2.86
NO_3_-N (mg N kg^−1^)	5.04

Treatments consisted of two residue placements (surface placement vs. incorporation into the soil) and three crop types (spring wheat, pea, and fallow [or no crop]) arranged in a completely randomized design with three replications. In order to match the residue and crop type, spring wheat residue was placed under spring wheat and fallow and pea residue under pea. Residues included nine-week old spring wheat and pea plants collected from the field without grains, chopped to 2 cm, and oven-dried at 60°C for 3 d. Fifteen grams of residues per pot (corresponding to 2.6 Mg ha^−1^ of residue found in the field) were either placed uniformly at the soil surface or incorporated into the soil by mixing the residue with the soil by hand. The surface placement of residue corresponded to the simulated no-tillage system in the field, although the soil was disturbed during collection, and incorporated residue to the simulated conventional tillage system. Spring wheat received 0.96 g N pot^−1^ as urea, similar to the recommended N fertilization rate (80 kg N ha^−1^) in the field, while pea received 0.11 g N pot^−1^ (or 9 kg N ha^−1^) while applying monoammonium phosphate as the P fertilizer. Half of 0.96 g N pot^−1^ was applied at planting and other half at four weeks later. Both spring wheat and pea also received P fertilizer (monoammonium phosphate) at 0.25 g P pot^−1^ (or 27 kg P ha^−1^) and K fertilizer (muriate of potash) at 0.50 g K pot^−1^ (or 29 kg K ha^−1^). No fertilizers were applied to the fallow treatment.

In July 2012, five spring wheat (cultivar Reeder) and pea (cultivar Majoret) seeds were planted per pot, except in the fallow treatment. At a height of 3 cm, seedlings were thinned to two plants per pot. In order to compensate for the water received as rainfall in the field, water was applied to all treatments in the greenhouse experiment to field capacity (0.25 m^3^ m^−3^) [21] at 300 to 500 mL pot^−1^. Water was applied at planting and at 3 to 7 d intervals thereafter, depending on soil water content (as determined by a soil water probe [TDR 300, Spectrum Technologies Inc., Aurora, IL] installed to a depth of 15 cm). Since measured amount of water was applied according to soil water content and crop demand, only a negligible amount of water was leached below the pot that was not determined. Herbicides and pesticides were applied to plants as needed. At 105 d after planting, shoot biomass including grains was harvested from the pot, washed with water, oven-dried at 60°C for 3 to7 d, and dry matter yield was determined. Because of the small amount of grain production, grains were also included in the shoot biomass. After crop harvest, soil from the entire pot was sieved to 2 mm to separate coarse residue and root fragments, which were picked by hand, washed with water, and oven-dried at 60°C for 3 to7 d to determine dry matter yields. A portion (100 g) of residue and root-free soil sample visible to the naked eye was collected from each pot, air-dried, and used for determinations of C and N fractions. The remaining soil samples were further washed in a nest of 1.0 and 0.5 mm sieves under a continuous stream of water to separate fine roots. Roots left in the sieves were picked using a tweezers, oven-dried at 60°C for 3 to7 d, and dry matter yield was determined. Total root biomass was determined by adding biomass of coarse and fine roots.

Shoot and root biomass and crop residues added to the soil at the initiation of the experiment and those (>2.00 mm) recovered from the soil at the end were ground to 1 mm and C and N concentrations (g kg^−1^) were determined with a high induction furnace C and N analyzer (LECO, St. Joseph, MI). Amounts of C and N in the residue added and recovered from the soil were determined by multiplying C and N concentrations by the weight of the soil in the pot. Carbon and N losses from the residue were determined as: Residue C and N losses (g kg^−1^) = (Residue C and N added – Residue C and N recovered)×1000/Residue C and N added. While determining the amount of C and N recovered in the residue, it was assumed that fine residue (<2.00 mm) was a part of soil organic matter.

### Field experiment

The field experiment was conducted using identical treatments, design, and replications as in the greenhouse from April to August 2012 near the place where soil samples were collected for the greenhouse experiment. As a result, soils were similar in both field and greenhouse experiments. The field site has mean monthly air temperature ranging from −8°C in January to 23°C in July and August. The mean annual precipitation (105-yr average) is 340 mm, 80% of which occurs during the crop growing season (April-October). Equivalent amounts of crop residues and fertilizers using the same treatments as in the greenhouse were applied to spring wheat, pea, and fallow in the field. Because the amount of residue applied was similar, the amounts of C and N added in residue to the soil were also identical in the greenhouse and field. Residues and fertilizers were placed at the soil surface in the no-till system and incorporated to a depth of 10 cm using tillage with a field cultivator in the conventional tillage system. Plot size was 12.2×6.1 m.

Spring wheat and pea were planted in April with a no-till drill at a spacing of 20.3 cm. Growing season weeds were controlled with selective post emergence herbicides appropriate for each crop. Contact herbicides were applied at postharvest and preplanting. Crops were grown under dryland condition receiving only precipitation without irrigation. In August, biomass yield of spring wheat and pea was determined from two 0.5 m^2^ areas outside yield rows within each plot and grain yield was determined by harvesting grains from a swath of 1.5 m×12.0 m using a combine harvester. Carbon and nitrogen concentrations in the grain and biomass were determined after oven drying subsamples at 55°C and using the C and N analyzer as above. Carbon and N contents (Mg C or N ha^−1^) in grain and biomass were determined by multiplying C and N concentrations by grain and biomass yields, respectively. Total aboveground biomass and C and N contents were determined by adding yields and C and N contents of grain and biomass.

Soil samples were collected from five random locations in central rows of the plot to a depth of 20 cm using a truck-mounted hydraulic probe (3.5 cm inside diameter). Samples were composited within a plot, air-dried, ground, and sieved to 2 mm for determining C and N concentrations. No attempts were made to collect the surface residue at soil sampling because of residue loss and contamination with soil and residue from one plot to another due to actions of wind and water. Therefore, residue C and N losses were not determined in the field.

### Soil carbon and nitrogen fractions measurements

The SOC concentration in the greenhouse and field soils were determined with a high induction furnace C and N analyzer as above after pretreating the soil with 5% H_2_SO_3_ to remove inorganic C [22]. The STN concentration was determined by using the analyzer without pretreating the soil with the acid. For determining POC and PON concentrations, 10 g soil sample was dispersed with 30 mL of 5 g L^−1^ sodium hexametaphosphate by shaking for 16 h and the solution was poured through a 0.053 mm sieve [19]. The solution and particles that passed through the sieve and contained mineral-associated and water-soluble C and N were dried at 50°C for 3 to 4 d and SOC and STN concentrations were determined by using the analyzer as above. The POC and PON concentrations were determined by the difference between SOC and STN in the whole-soil and that in the particles that passed through the sieve after correcting for the sand content.

The PCM and PNM concentrations in air-dried soils were determined by the modified method of Haney et al. [23]. Two 10 g soil subsamples were moistened with water at 50% field capacity [21] and placed in a 1 L jar containing beakers with 4 mL of 0.5 mol L^-1^ NaOH to trap evolved CO_2_ and 20 mL of water to maintain high humidity. Soils were incubated in the jar at 21°C for 10 d. At 10 d, the beaker containing NaOH was removed from the jar and PCM was determined by measuring CO_2_ absorbed in NaOH, which was back-titrated with 1.5 mol L^−1^ BaCl_2_ and 0.1 mol L^−1^ HCl. One beaker containing soil was removed from the jar and extracted with 100 mL of 2 mol L^−1^ KCl for 1 h. The NH_4_-N and NO_3_-N concentrations in the extract were determined by using the autoanalyzer (Lachat Instrument, Loveland, CO). The PNM was calculated as the difference between the sum of NH_4_-N and NO_3_-N concentrations in the soil before and after incubation.

The other beaker containing moist soil and incubated for 10 d (used for PCM determination above) was used for determining MBC and MBN concentrations by the modified fumigation–incubation method for air-dried soils [24]. The moist soil was fumigated with ethanol-free chloroform for 24 h and placed in a 1 L jar containing beakers with 2 mL of 0.5 mol L^−1^ NaOH and 20 mL water. As with PCM, fumigated moist soil was incubated for 10 d and CO_2_ absorbed in NaOH was back-titrated with BaCl_2_ and HCl. The MBC was calculated by dividing the amount of CO_2_–C absorbed in NaOH by a factor of 0.41 [25] without subtracting the values from the nonfumigated control [24]. For MBN, the fumigated–incubated sample at 10 d was extracted with 100 mL of 2 mol L^-1^ KCl for 1 h and NH_4_-N and NO_3_-N concentrations were determined by using the autoanalyzer as above. The MBN was calculated by the difference between the sum of NH_4_-N and NO_3_-N concentrations in the sample before and after fumigation–incubation and divided by a factor of 0.41 [25], [26]. The NH_4_-N and NO_3_-N concentrations determined in the nonfumigated–nonincubated samples were used as available fractions of N.

### Data analysis

Data for C and N contents in crop biomass and residue and soil C and N fractions were analyzed by using the MIXED model of SAS [27]. Treatment was considered as the fixed effect and replication as the random effect. Means were separated by using the least square means test when treatments and interactions were significant [27]. Statistical significance was evaluated at *P*≤0.05, unless otherwise stated.

## Results

### Greenhouse experiment

#### Shoot and root biomass yields and carbon and nitrogen contents

Shoot and root biomass yields and C and N contents varied among residue placements and crop types (Table 2). Interaction between residue placement and crop types on these parameters was not significant. Shoot and root biomass yields and C and N contents were greater in surface placement than incorporation of residue into the soil. Shoot biomass yield and C and N contents were also greater in wheat than in pea. Because of the negligible amount of roots, root biomass yield and C and N contents in pea were not determined. Absence of plants in the fallow also resulted in non-existence of crop data in this treatment. The coefficient of variation (CV) for crop parameters ranged from 38.2 to 62.5%.

**Table 2:** Effects of residue placement and crop type on crop shoot (grains+leaves+stems) and root biomass C and N contents in the greenhouse.

Residue placement	Crop type	Shoot biomass	Root biomass	Shoot biomass C	Root biomass C	Shoot biomass N	Root biomass N	Total biomass C	Total biomass N
		--------g pot^−1^------		--------g C pot^−1^------		--------g Npot^−1^------		g C pot^−1^	g N pot^−1^
Incorporated		4.51b^a^	2.37b	1.83b	0.74b	0.14b	0.05b	2.91b	0.25b
Surface		7.41a	6.14a	3.07a	1.74a	0.24a	0.11a	5.55a	0.44a
	Pea	4.55b	-----b	1.91b	-----	0.12b	-----	1.91b	0.12b
	Wheat	7.36a	4.25	3.00a	1.24	0.26a	0.08	4.23a	0.34a
CV (%)^c^		42.4	61.9	42.4	56.4	55.0	62.5	40.4	38.2
Significance									
Residue placement (R)		*	-----	*	*	**	*	*	*
Crop species (C)		*	*	*	-----	**	-----	*	-----
R×C		NS^d^	NS	NS	-----	NS	-----	-----	-----

*Significant at *P* = 0.05.

**Significant at *P* = 0.01.

a Numbers followed by different letters within a column in a set are significantly different at *P*≤0.05 by the least square means test.

b Non-measurable values due to negligible amount of root biomass.

c Coefficient of variation.

d Not significant.

#### Residue carbon and nitrogen losses

Total amounts of C and N added through residue application and leaf fall and those recovered in coarse fractions (>2 mm) after crop harvest varied with residue placements and crop types, with the significant residue placement × crop type interaction for C and N recovered in the residue (Table 3). Although the amount of residue applied was similar in all treatments (15 g of wheat or pea residue pot^−1^), differences in C and N concentrations between residues and those added through leaf fall during crop growth varied residue C and N additions among treatments. Averaged across crop types, residue C addition was greater in surface placement than incorporation of residue into the soil. Averaged across residue placements, residue C addition was greater under wheat than pea or fallow, but residue N addition was greater under pea than wheat or fallow. Residue C recovery was greater in surface placement under wheat and fallow than surface placement under pea and incorporation under fallow. Residue N recovery was also greater in surface placement under wheat and fallow than surface placement under pea and incorporation under fallow and wheat. Averaged across crop types, residue N recovery was greater in surface placement than incorporation of residue into the soil. Averaged across residue placements, residue C recovery was greater under wheat than pea. The coefficient of variation for residue C and N addition and recovery varied from 7.2 to 17.8%.

**Table 3:** Effects of residue placement and crop type on residue C and N addition, recovered in coarse fragments (>2 mm), and losses during the crop growing period in the greenhouse.

Residue placement	Crop type	Crop residue					
		C added^a^	N added^a^	C recovered	N recovered	C loss	N loss
		g C pot^−1^	g N pot^−1^	g C pot^−1^	g N pot^−1^	g kg^−1^	g kg^−1^
Incorporated	Fallow	7.80a^b^	0.40a	3.84b	0.23b	508a	438b
	Pea	7.80a	0.64a	4.24ab	0.26ab	457ab	588a
	Wheat	8.76a	0.44a	4.89ab	0.24b	430ab	456b
Surface	Fallow	7.80a	0.40a	5.16a	0.31a	339b	213d
	Pea	7.80a	0.64a	3.82b	0.23b	511a	640a
	Wheat	8.92a	0.46a	4.95a	0.31a	446ab	325c
CV (%)^c^		7.2	17.8	15.4	16.5	143	316
Means							
Incorporated		8.07b	0.49a	4.32a	0.24b	465a	494a
Surface		8.17a	0.50a	4.64a	0.28a	432a	393b
	Fallow	7.80b	0.40b	4.50ab	0.27a	423a	326b
	Pea	7.80b	0.64a	4.02b	0.25a	484a	614a
	Wheat	8.76a	0.45b	4.92a	0.28a	438a	390b
Significance							
Residue placement (R)		*	NS^d^	NS	*	NS	**
Crop species (C)		***	***	*	NS	*	***
R×C		NS	NS	*	*	*	**

*Significant at *P* = 0.05.

**Significant at *P* = 0.01.

*** Significant at *P* = 0.001.

a Includes C and N added from the residue application and leaf fall.

b Numbers followed by different letters within a column in a set are significantly different at *P*≤0.05 by the least square means test.

c Coefficient of variation.

d Not significant.

Residue C and N losses also varied with residue placements and crop species, with the significant residue placement × crop species interaction (Table 3). Residue C loss was greater in surface placement under pea and incorporation under fallow than surface placement under fallow. Residue N loss was in the order: surface placement and incorporation under pea > incorporation under wheat and fallow > surface placement under wheat > surface placement under fallow. Averaged across crop types, residue N loss was greater in residue incorporation than surface placement. Averaged across residue placements, residue N loss was greater under pea than under fallow and wheat. The coefficient of variation for residue C and N losses varied from 14.3 to 31.6%.

#### Soil carbon and nitrogen fractions

The SOC, POC, and PCM concentrations varied among residue placements and crop types (Table 4). Averaged across crop types, SOC and PCM were greater in surface placement than incorporation of the residue into the soil. Averaged across residue placements, SOC was greater under wheat than pea and fallow and POC was greater under wheat than pea. The MBC was not influenced by treatments. The coefficient of variation for soil C fractions ranged from 2.6 to 14.3%.

**Table 4:** Effects of residue placement and crop type on soil organic C (SOC), total N (STN), particulate organic C and N (POC and PON), potential C and N mineralization (PCM and PNM), microbial biomass C and N (MBC and MBN), and NH_4_-N and NO_3_-N concentrations in the greenhouse.

Residue placement	Crop type	SOC	POC	PCM	MBC	STN	PON	PNM	MBN	NH_4_-N	NO_3_-N
		--------- g C kg^−1^------		-------- mg C kg^−1^------		------ g N kg^−1^------		--------------------------- mg N kg^−1^----------------			
Incorporated		12.0b^a^	3.28a	11.6b	128.0a	1.29a	0.33a	5.80b	50.8a	1.14b	11.8a
Surface		12.3a	3.28a	14.4a	143.8a	1.31a	0.31a	9.56a	57.5a	1.65a	16.5a
	Fallow	12.0b	3.26ab	11.4a	118.9a	1.30ab	0.32a	9.36a	46.1b	1.44a	23.7a
	Pea	12.0b	3.18b	13.1a	144.0a	1.26b	0.29a	9.68a	56.3ab	1.38a	12.8ab
	Wheat	12.4a	3.40a	14.5a	145.1a	1.34a	0.34a	5.98b	97.6a	1.36a	6.1b
CV (%)^b^		2.6	11.8	14.3	13.3	4.6	15.9	14.2	14.3	14.8	13.6
Significance											
Residue placement (R)		**	NS^c^	*	NS	NS	NS	*	NS	*	NS
Crop species (C)		**	*	NS	NS	*	NS	*	*	NS	*
R×C		NS	NS	NS	NS	NS	NS	NS	NS	NS	NS

**Soil samples were collected at the 0–20 cm depth in the field and used for the greenhouse experiment.**

*Significant at *P* = 0.05.

**Significant at *P* = 0.01.

a Numbers followed by different letters within a column in a set are significantly different at *P*≤0.05 by the least square means test.

b Coefficient of variation.

c Not significant.

The STN, PNM, MBN, NH_4_-N, and NO_3_-N concentrations also varied among residue placements and crop types (Table 4). Averaged across crop types, PNM and NH_4_-N were greater in surface placement than incorporation of residue into the soil. Averaged across residue placements, STN was greater under wheat than pea and MBN was greater under wheat than fallow. In contrast, PNM was greater under pea and fallow than wheat and NO_3_-N was greater under fallow than wheat. The PON was not influenced by treatments. The coefficient of variation for soil N fractions ranged from 4.6 to 15.9%.

### Field experiment

Aboveground total crop biomass yield and C and N contents varied with crop types (Table 5). Averaged across tillage practices, crop biomass yield and C content were greater in wheat than pea, but the trend reversed for N content. Tillage and its interaction with crop type were not significant for crop biomass yield and C and N contents. The coefficient of variation for crop biomass yield and C and N contents ranged from 28.1 to 41.9%.

**Table 5:** Effects of residue placement and crop type on crop aboveground biomass (grains+stems+leaves) yield, C and N contents, and soil organic C (SOC), total N (STN), particulate organic C and N (POC and PON), potential C and N mineralization (PCM and PNM), microbial biomass C and N (MBC and MBN), and NH_4_-N and NO_3_-N concentrations at the 0–20 cm depth in the field.

Tillage^a^	Crop type	Crop biomass yield	Crop C content	Crop N content	SOC	POC	PCM	MBC	STN	PON	PNM	MBN	NH_4_-N	NO_3_-N
		Mg ha^−1^	Mg C ha^-1^	kg N ha^−1^	----- g C kg^−1^-----		----- mg C kg^−1^----		----- g N kg^−1^-----		------------------- mg N kg^−1^-----------------			
CT		4.91a^b^	2.06a	59.6a	11.0a	2.56a	45.1a	114.4a	1.33a	0.24a	3.21a	12.6b	3.05b	4.54a
NT		5.08a	2.11a	65.3a	110.a	2.56a	56.8a	122.9a	1.37a	0.28a	4.28a	19.6a	3.82a	2.08b
	Fallow	------c	------	------	10.6a	2.55a	45.61a	111.4b	1.30a	0.25a	2.85b	14.2a	2.93a	3.36a
	Pea	4.68b	1.87b	72.3a	11.4a	2.56a	51.0a	118.6ab	1.42a	0.27a	5.56a	15.5a	3.43a	2.87a
	Wheat	5.31a	2.18a	52.6b	11.0a	2.57a	56.0a	126.0a	1.34a	0.26a	2.83b	18.6a	3.93a	3.71a
CV (%)		28.1	30.0	41.9	8.0	18.7	27.1	28.9	9.3	18.4	36.4	36.7	27.9	25.0
Significance														
Tillage (T)		NS^d^	NS	NS	NS	NS	NS	NS	NS	NS	NS	*	*	*
Crop species (C)		*	*	**	NS	NS	NS	*	NS	NS	*	NS	NS	NS
T×C		NS	NS	NS	NS	NS	NS	NS	NS	NS	NS	NS	NS	NS

*Significant at *P* = 0.05.

**Significant at *P* = 0.01.

a Tillage are CT, conventional tillage; and NT, no-tillage.

b Numbers followed by different letters within a column in a set are significantly different at *P*≤0.05 by the least square means test.

c Crop absent in the fallow.

d Not significant.

Soil MBN, NH_4_-N, and NO_3_-N concentrations varied with tillage practices and MBC and PNM varied with crop types (Table 5). Averaged across crop types, MBN and NH_4_-N were greater in no-tillage than conventional tillage, but NO_3_-N was greater in conventional tillage than no-tillage. Averaged across tillage practices, MBC was greater under wheat than fallow and PNM was greater under pea than wheat and fallow. Tillage, crop type, and their interaction were not significant for SOC, POC, PCM, STN, and PON. The coefficient of variations for soil C and N fractions ranged from 8.0 to 36.7%.

## Discussion

Enhanced soil water conservation due to mulch action of the residue at the soil surface [28] may have increased shoot and root biomass yields and C and N contents in surface placement compared to incorporation of residue into the soil in the greenhouse (Table 2). It has been reported that surface placement of residue in the no-till system increased spring wheat yield compared to residue incorporation in the conventional till system [3], [15]. In our field experiment, crop biomass yield and C and N contents, however, were not influenced by tillage (Table 5). It may be possible that wheat and pea residues applied by hand at the soil surface were more uniformly distributed in the greenhouse than in the field where residues were distributed by a machine sprayer. As a result, soil water was probably conserved more, resulting in increased crop yield and C and N contents with the surface placement than incorporation of residue in the greenhouse compared to the field.

Differences in the amount of N fertilizer applied and N fixation capacity may have resulted in variation in crop biomass yields and C contents among crop species in the greenhouse and the field (Tables 2 and 5). Higher amount of N fertilizer application may have increased biomass yield and C and N contents in wheat than pea in the greenhouse. Higher amount of N fertilizer application also may have increased biomass yield and C content in wheat and pea, but greater N fixation may have increased N content in pea than wheat in the field [14], [28]. Grain and biomass yields are usually greater in wheat which receives N fertilizer than pea which receives no N fertilizer due to increased water-use efficiency, but higher N concentration due to increased atmospheric N fixation can increase N content in pea than wheat [10], [15], [28]. The fact that different trends in N content in pea vs. wheat occurred in the field and the greenhouse was probably related to root growing soil volume. It may be possible that roots exploited greater soil volume that resulted in increased N fixation by pea and therefore increased its N content in the field compared to the greenhouse where plants were grown in a limited soil volume in the pot.

Greater residue input due to higher biomass yield may have increased residue C addition in surface placement than incorporation of residue into the soil or increased under wheat than pea or fallow in the greenhouse (Tables 2 and 3). In contrast, higher N concentration may have increased residue N addition under pea than wheat or fallow. Greater C and N recovered in the residue placed at the soil surface under wheat and fallow were probably due to reduced mineralization of wheat residue as a result of its higher C/N ratio than pea residue. While surface placement of residue reduces its contact with soil microorganisms that result in reduced mineralization [29], [30], increased mineralization of pea residue due to its lower C/N ratio may have resulted in reduced C and N recovery in the residue placed at the soil surface under pea. Residues of legumes, such as pea with lower C/N ratio, decompose more rapidly than those of nonlegumes, such as wheat with higher C/N ratio [12]. When incorporated into the soil, residue C and N recovery were lower under fallow and wheat. As a result, C and N losses were higher in surface placement of residue under pea or residue incorporation under pea and fallow than the other treatments. It may be possible that some of C and N lost from the residue converted into soil C and N fractions, as discussed below.

Reduced mineralization of residue may have increased SOC, PCM, PNM, and NH_4_-N concentrations in surface placement than incorporation of residue into the soil in the greenhouse (Table 4). Similar increases in MBN and NH_4_-N concentrations in no-tillage compared to conventional tillage were found in the field (Table 5). Several researchers [5], [16], [31], [32], [33] have reported greater SOC, POC, MBC, PCM, PNM, and MBN in surface residue placement in the no-tillage system than residue incorporation into the soil in the conventional tillage system. Increased N mineralization due to residue incorporation, however, may have increased NO_3_-N concentration in conventional tillage than no-tillage in the field.

Higher C and N substrate availability due to increased yield probably increased SOC, POC, STN, and MBN under wheat than under pea or fallow in the greenhouse (Table 4) or increased MBC under wheat than fallow in the field (Table 5). Root biomass C, residue C addition (Tables 2 and 3), and amount of applied N fertilizer were greater in wheat than pea or fallow. Similar results probably occurred in the field, since treatments were identical in the greenhouse and the field and crop biomass C was higher in wheat than pea in the field (Table 5). Rhizodeposit C released by roots can increase microbial biomass and activity and soil C storage [34]. Liebig et al. [35] also found higher MBC under spring wheat than under fallow. In contrast, greater PNM and NO_3_-N under pea and fallow than wheat in the greenhouse were probably either due to increased mineralization of pea residue as a result of its lower C/N ratio than wheat residue [12] or to greater mineralization of soil and wheat residue as a result of enhanced microbial activity from higher soil temperature and water content and absence of plants to uptake N under fallow [11], [13], [36]. Since residue N loss was greater under pea than wheat and fallow (Table 3), part of N from pea residue may have contributed to increased PNM and NO_3_-N concentrations under pea. Similar result of increased PNM under pea than wheat and fallow was also found in the field, since crop biomass N was greater in pea than wheat (Table 5).

Comparison of soil C and N fractions at the beginning and end of the experiment due to residue placement (Tables 1 and 4) showed that SOC increased by 4.2%, PCM by 55.7%, and PNM by 6.1% with surface residue placement in the greenhouse. Corresponding values in SOC, PCM, and PNM with residue incorporation were 1.7, 25.4, and −35.1%, respectively. In the field, MBN reduced by 71.5% in no-tillage and 81.7% in conventional tillage from the beginning to the end of the experiment. This shows that residue placement at the surface either increased soil C and N fractions in the greenhouse or reduced their losses in the field within a crop growing season compared to residue incorporation. Since soil NH_4_-N and NO_3_-N concentrations vary seasonally due to N mineralization from crop residue and soil, N fertilization, crop N uptake, and N losses due to leaching, volatilization, and denitrification [10], [15], variations in their levels from the beginning to the end of the experiment were not taken into account.

Among crop types, SOC increased by 5.1%, POC by 6.9%, STN by 3.9%, and MBN by 41.4%, but PNM decreased by 33.1% under wheat from the beginning to the end of the experiment in the greenhouse. In the field, MBC increased by 7.1%, but PNM decreased by 68.3% under wheat during this period. The corresponding increases in SOC, POC, STN, and MBN or decrease in PNM during this period were lower under pea and fallow. This suggests that wheat increased more soil C and N fractions, except PNM, than pea or fallow due to increased substrate availability from root and rhizodeposition and/or to slow decomposition of wheat than pea residue due to differences in residue quality (e.g. C/N ratio). The greater PNM under pea than wheat or fallow was due to increased N contribution from its residue (Table 5).

When the greenhouse and field experiments were compared, trends in changes in soil C and N fractions due to treatments within a crop growing season were similar. However, greater changes in labile than nonlabile C and N fractions occurred more in the greenhouse than in the field. Furthermore, the coefficient of variations in soil C and N fractions were lower in the greenhouse (2.6 to 15.9%) than in the field (8.0 to 36.7%) (Tables 4 and 5). This indicates that soil C and N fractions changed more readily but with lower variability with management practices within a crop growing season when soil and environmental conditions are controlled in the greenhouse than in field where soil heterogeneity often results in non-significant differences among treatments in these fractions [16], [18], [30]. Use of disturbed soil in the greenhouse vs. undisturbed (especially in the no-till system) in the field also may have an influence on differences in changes in soil C and N fractions between the two experiments. The greater changes in labile than nonlabile C and N fractions as influenced by management practices within a short period in the greenhouse and the field suggests that labile C and N fractions are better indicators of changes in soil organic matter quality than nonlabile fractions, a case similar to that reported by various researchers [10], [11], [13], [16], [30]. The fact that more changes in labile than nonlabile C and N fractions occurred in the greenhouse than in the field suggests that better measurements of changes in soil organic matter due to management practices within a short period can be observed when soil and environmental conditions are controlled. Greater levels of most soil C and N fractions in the greenhouse than in field was probably a result of increased turnover rate plant C and N into soil C and N, because disturbed soil was used in the greenhouse and environmental condition for microbial transformation was more favorable in the greenhouse than the field.

Greenhouse study provided more information on plant and residue parameters, such as measurement of root biomass and C and N contents and residue C and N losses, which cannot be measured easily in the field. This resulted in the measurement of turnover rate of plant C and N into soil C and N in the greenhouse, a fact that was absent in the field. Because of greater changes in soil C and N fractions, greenhouse study provided a more robust method of evaluating C and N cycling and soil quality within a short period of time as affected by management practices than the field experiment. Such changes can also be measured in the field but it may take longer time. While all results from the greenhouse study may not be readily applied in the field, some information, such as root biomass and residue C and N losses, measured in the greenhouse can be extrapolated to the field condition. The effects of short-term study in the greenhouse can be useful to predict the long-term impact of management practices on soil C and N fractions in the field.

## Conclusions

Crop yields, residue C and N losses, and soil C and N fractions varied with residue placement and crop types in the greenhouse and the field. Surface placement of residue increased crop yields, residue C and N losses, and enhanced SOC, PCM, MBN, and NH_4_-N concentrations, but residue incorporation increased PNM and NO_3_-N concentrations. Similarly, spring wheat had higher yield and increased SOC, POC, MBC, STN, and MBN than pea or fallow, but pea had higher N content and increased PNM than wheat or fallow. Placing nonlegume residue at the soil surface using no-tillage can increase soil C and N sequestration and microbial biomass and activity that can improve soil health and quality. Using this practice, producers can claim for C credit. Incorporation of legume and nonlegume residues into the soil using conventional tillage can increase N mineralization and availability which can reduce N fertilization rate to succeeding crops, but can degrade soil quality due to reduced organic matter and increased erosion. Although soil labile C and N fractions changed more readily than nonlabile fractions within a crop growing season both in the greenhouse and field, greater changes in labile than nonlabile fractions occurred with reduced variability more in the greenhouse than in the field. Results suggest that greenhouse study provided a more robust measurement of crop growth and changes in soil C and N fractions within a short period as influenced by management practices than the field experiment. Longer time will be probably needed in the field to obtain results similar to those in the greenhouse. Additional information, such as root growth, residue C and N losses, turnover of plant C and N to soil C and N, and results of short-term study on soil C and N fractions as influenced by management practices in the greenhouse can be used to predict the long-term impact in the field.

## References

[1] Lal R, Kimble JM, Stewart BA (1995) World soils as a source or sink for radiatively-active gases. In: Lal R, editor, Soil management and greenhouse effect. Advances in soil science. CRC Press, Boca Raton, FL, pp. 1–8

[2] Paustian K, Robertson GP, Elliott ET (1995) Management impacts on carbon storage and gas fluxes in mid-latitudes cropland. In: Lal R, editor, Soils and global climate change. Advances in soil science. CRC Press, Boca Raton, FL, USA, pp. 69–83.

[3] Halvorson AD, Peterson GA, Reule CA (2002a) Tillage system and crop rotation effects on dryland crop yields and soil carbon in the central Great Plains. Agron J 94: 1429–1436.

[4] Sherrod LA, Peterson GA, Westfall DG, Ahuja LR (2003) Cropping intensity enhances soil organic carbon and nitrogen in a no-till agroecosystem. Soil Sci Soc Am J 67: 1533–1543.

[5] Sainju UM, Caesar-TonThat T, Lenssen AW, Evans RG, Kolberg R (2007) Long-term tillage and cropping sequence effects on dryland residue and soil carbon fractions. Soil Sci Soc Am J 71: 1730–1739.

[6] Rasmussen PE, Allmaras RR, Rhoade CR, Roager NC Jr (1980) Crop residue influences on soil carbon and nitrogen in a wheat-fallow system. Soil Sci Soc Am J 44: 596–600.

[7] Peterson GA, Halvorson AD, Havlin JL, Jones OR, Lyon DG, Tanaka DL (1998) Reduced tillage and increasing cropping intensity in the Great Plains conserve soil carbon. Soil Tillage Res 47: 207–218.

[8] Bauer A, Black AL (1994) Quantification of the effect of soil organic matter content on soil productivity. Soil Sci Soc Am J 58: 185–193.

[9] Ghidey F, Alberts EE (1993) Residue type and placement effects on decomposition: Field study and model evaluation. Trans ASAE 36: 1611–1617.

[10] Sainju UM, Lenssen AW, Caesar-Tonthat T, Waddell J (2006b) Tillage and crop rotation effects on dryland soil and residue carbon and nitrogen. Soil Sci Soc Am J 70: 668–678.

[11] Halvorson AD, Wienhold BJ, Black AL (2002b) Tillage, nitrogen, and cropping system effects on soil carbon sequestration. Soil Sci Soc Am J 66: 906–912.

[12] Kuo S, Sainju UM, Jellum EJ (1997) Winter cover cropping influence on nitrogen in soil. Soil Sci Soc Am J 61: 1392–1399.

[13] Sainju UM, Lenssen AW, Caesar-TonThat T, Waddell J (2006a) Carbon sequestration in dryland soils and plant residue as influenced by tillage and crop rotation. J Environ Qual 35: 1341–1349. 16825454 10.2134/jeq2005.0131

[14] Miller PR, McConkey B, Clayton GW, Brandt SA, Staricka JA, Johnston AM, Lafond GP, Schatz BG, Baltensperger DD, Neill KE (2002) Pulse crop adaptation in the northern Great Plains. Agron J 94: 261–272.

[15] Sainju UM, Lenssen AW, Caesar-TonThat T, Evans RG (2009) Dryland crop yields and soil organic matter as influenced by long-term tillage and cropping sequence. Agron J 101: 243–251.

[16] Franzluebbers AJ, Hons FM, Zuberer DA (1995) Soil organic carbon, microbial biomass, and mineralizable carbon and nitrogen in sorghum. Soil Sci Soc Am J 59: 460–466.

[17] Bezdicek DF, Papendick DF, Lal R (1996) Introduction: Importance of soil quality to health and sustainable land management. In: Doran JW, Jones AJ, editors, Methods of assessing soil quality, Spec. Publ. 49, Soil Science Society of America, Madison, USA, pp. 1–18.

[18] Franzluebbers AJ, Arshad MA (1997) Soil microbial biomass and mineralizable carbon of water-stable aggregates. Soil Sci Soc Am J 67: 1090–1097.

[19] Cambardella CA, Elliott ET (1992) Particulate soil organic matter changes across a grassland cultivation sequence. Soil Sci Soc Am J 56: 777–783.

[20] Six J, Elliott ET, Paustian K (1999) Aggregate and soil organic matter dynamics under conventional and no-tillage systems. Soil Sci Soc Am J 63: 1350–1358.

[21] Pikul JL Jr, Aase JK (2003) Water infiltration and storage affected by subsoiling and subsequent tillage. Soil Sci Soc Am J 67: 859–866.

[22] Nelson DW, Sommers LE (1996) Total carbon, organic carbon, and organic matter. In: Sparks DL, editor, Methods of soil analysis. Part 3. Chemical method. SSSA Book Ser. 5. Soil Science Society of America, Madison, pp. 961–1010.

[23] Haney RL, Franzluebbers AJ, Porter EB, Hons FM, Zuberer DA (2004) Soil carbon and nitrogen mineralization: Influence of drying temperature. Soil Sci Soc Am J 68: 489–492.

[24] Franzluebbers AJ, Haney RL, Hons FM, Zuberer DA (1996) Determination of microbial biomass and nitrogen mineralization following rewetting of dried soil. Soil Sci Soc Am J 60: 1133–1139.

[25] Voroney RP, Paul EA (1984) Determination of k_C_ and k_N_ *in situ* for calibration of the chloroform fumigation-incubation method. Soil Biol Biochem 16: 9–14.

[26] Brookes PC, Landman A, Pruden G, Jenkinson DJ (1985) Chloroform fumigation and the release of soil nitrogen: A rapid direct-extraction method to measure microbial biomass nitrogen in soil. Soil Biol Biochem 17: 937–942.

[27] Littell RC, Milliken GA, Stroup WW, Wolfinger RR (1996) SAS system for mixed models. SAS Institute Inc., Cary, NC, USA.

[28] Lenssen AW, Johnson GD, Carlson GR (2007) Cropping sequence and tillage system influences annual crop production and water use in semiarid Montana. Field Crops Res 100: 32–43.

[29] Coppens F, Garnier P, de Gryze P, Merckx R, Recous S (2006) Soil moisture, carbon and nitrogen dynamics following incorporation and surface application of labelled crop residues in soil columns. Europ J Soil Sci 57: 894–905.

[30] Giacomini SJ, Recous S, Mary B, Aita C (2007) Simulating the effects of nitrogen availability, straw particle size and location in soil on carbon and nitrogen mineralization. Plant Soil 301: 289–301.

[31] Malhi SS, Lemke R (2007) Tillage, crop residue and nitrogen fertilizer effects on crop yield, nutrient uptake, soil quality and greenhouse gas emissions in the second 4-yr rotation cycle. Soil Tillage Res 96: 269–283.

[32] Wright AL, Hons FM, Lemon RG, MacFarland ML, Nichols RL (2008) Microbial activity and soil carbon sequestration for reduced and conventional tillage cotton. Appl Soil Ecol 38: 168–173.

[33] Lupwayi NZ, Lafond GP, Ziadi N, Grant CA (2012) Soil microbial response to nitrogen fertilizer and tillage in barley and corn. Soil Tillage Res 118: 139–146.

[34] Lu Y, Watanabe A, Kimura M (2002) Contribution of plant-derived carbon to soil microbial biomass dynamics in a paddy rice microcosm. Biol Fertil Soils 36: 136–142.

[35] Liebig MA, Tanaka DL, Wienhold BJ (2004) Tillage and cropping effects on soil quality indicators in the northern Great Plains. Soil Tillage Res 78: 131–141.

[36] Kuzyakov Y, Domanski G (2000) Carbon input by plants into the soil: Review. J. Plant Nutri Soil Sci 163: 421–431.

